# Adaptive habitat biogeography-based optimizer for optimizing deep CNN hyperparameters in image classification

**DOI:** 10.1016/j.heliyon.2024.e28147

**Published:** 2024-03-21

**Authors:** Jiayun Xin, Mohammad Khishe, Diyar Qader Zeebaree, Laith Abualigah, Taher M. Ghazal

**Affiliations:** a School of Mechanical, Electrical and Information Engineering, Shandong University, Weihai, 264209, Shandong, China; b Department of Electrical Engineering, Imam Khomeini Marine Science University, Nowshahr, Iran; c Center for Artificial Intelligence Applications, Yuan Ze University, Taiwan; d Information Technology Department, Technical College of Duhok, Duhok Polytechnic University, Duhok, Iraq; e Hourani Center for Applied Scientific Research, Al-Ahliyya Amman University, Amman, 19328, Jordan; f Computer Science Department, Al al-Bayt University, Mafraq, 25113, Jordan; g Artificial Intelligence and Sensing Technologies (AIST) Research Center, University of Tabuk, Tabuk, 71491, Saudi Arabia; h MEU Research Unit, Middle East University, Amman, 11831, Jordan; i Department of Electrical and Computer Engineering, Lebanese American University, Byblos, 13-5053, Lebanon; j School of Engineering and Technology, Sunway University Malaysia, Petaling Jaya, 27500, Malaysia; k Centre for Cyber Physical Systems, Computer Science Department, Khalifa University, United Arab Emirates; l Center for Cyber Security, Faculty of Information Science and Technology, Universiti Kebangsaan Malaysia (UKM), 43600, Bangi, Selangor, Malaysia; m Applied Science Research Center, Applied Science Private University, Amman, 11937, Jordan

**Keywords:** Digital image classification, Deep convolutional neural networks, Biogeography-based optimizer, Adaptive habitat

## Abstract

Deep Convolutional Neural Networks (DCNNs) have shown remarkable success in image classification tasks, but optimizing their hyperparameters can be challenging due to their complex structure. This paper develops the Adaptive Habitat Biogeography-Based Optimizer (AHBBO) for tuning the hyperparameters of DCNNs in image classification tasks. In complicated optimization problems, the BBO suffers from premature convergence and insufficient exploration. In this regard, an adaptable habitat is presented as a solution to these problems; it would permit variable habitat sizes and regulated mutation. Better optimization performance and a greater chance of finding high-quality solutions across a wide range of problem domains are the results of this modification's increased exploration and population diversity. AHBBO is tested on 53 benchmark optimization functions and demonstrates its effectiveness in improving initial stochastic solutions and converging faster to the optimum. Furthermore, DCNN-AHBBO is compared to 23 well-known image classifiers on nine challenging image classification problems and shows superior performance in reducing the error rate by up to 5.14%. Our proposed algorithm outperforms 13 benchmark classifiers in 87 out of 95 evaluations, providing a high-performance and reliable solution for optimizing DNNs in image classification tasks. This research contributes to the field of deep learning by proposing a new optimization algorithm that can improve the efficiency of deep neural networks in image classification.

## 1 Introduction

DCNNs have indicated their outstanding advantages in Digital Image Processing (DIP) tasks [1], such as Autonomous Underwater Vehicle (AUV) Systems [2], cognitive and social networks [3,4], traffic management [5], reverse auctions [6], epidemic model detection [7], predictive control [8], recognition [9], detection [10], and image classification [11,12]. DCNNs are initially motivated by the cat visual cortex’s computational model differentiating signal-related and processing vision tasks [13]. Since Yann LeCun introduced LetNet-5 in 1989 [14], in which its connection weights are tuned by the canonical back-propagation method, different DCNNs have been introduced, including ResNet [15], GoogleNet [16], DenseNet [17], SENet [18], Sketch2Photo [19], RayMVSNet++ [20], and VGGNet [21]. When compared to the standard models for image classification problems, these models significantly increased the classification rate [22]. These deep networks are different in terms of structure and connection weights. Many efforts, such as transfer learning, self-supervised learning, fuzzing with invocation ordering [23,24], fusion models [25], hierarchical models [26], adapting feature selection algorithms [27], subspace random optimization [28], multi-modal [29], and multi-label [30] techniques have improved the performance of these models [31].

The DCNN’s primary block sets are convolutional, pooling, and fully connected layers [32]. DCNNs can effectively solve various DIP problems by tuning the mentioned layers properly in models [33]. The efficiency of DCNNs significantly depends on the parameters, including the number of pooling and convolutional layers, the filter size, the stride, the learning rate, the number of hidden units, the batch size, the activation function type, the type of padding, the dropout probability, the number of epochs, the regularization technique, the number of filters, etc [34]. These parameters are referred to as DCNN’s hyperparameters. The hyperparameters' tuning referred to the task of determining the right values of hyperparameters for a particular problem [35]. The optimization of hyperparameters is an Nondeterministic Polynomial (NP)-hard problem, which is one of the primary challenges confronting DCNN’s training [36]. As reported by the literature review, most methods focused on increasing classification accuracy by modifying or expanding DCNN’s structure [37]. However, only a few methods addressed hyperparameters' optimization tasks [38].

The most well-known hyperparameter optimization methods are random search [39], grid search [40], and Bayesian search [38,41]. Grid search utilizes all probable hyperparameter combinations, and each of the hyperparameter combinations denotes a machine-learning paradigm. Consequently, *N* combinations demonstrate that *N* machine learning paradigms identify the best model for performance. Therefore, grid search is costly in terms of both time and space complexity [42].

On the other hand, a random search randomly selects the hyperparameters’ combination, which is quite computationally cheap. However, the main shortcoming of this technique is neglecting the previously obtained results. This defect means it does not learn from prior experiments. Therefore, it is possible to search with identical hyperparameters [43].

Bayesian search is based on the Bayes principle, where compared to the posterior probability distribution, the prior probability distribution is precisely proportional to the prior probability distribution. This proportion means that prior probability distribution can be considered to be expert knowledge, leading to augmenting the experience produced by training data [44].

The three search methods mentioned above need much knowledge and time [45]. In order to adjust a balance between accuracy and comprehensiveness on the one hand and spatial and temporal complexity on the other, we propose a meta-heuristic algorithm [46] for the hyperparameters’ tuning and structure design by applying the BBO algorithm [47]. Contrary to random search, the meta-heuristic algorithm learns in each iteration so that searching agents move toward superior hyperparameters [48]. As the optimization of DCNN hyperparameters is an NP-hard task, satisfying results could be obtained by employing metaheuristic-based approaches [49,50].

Many metaheuristic algorithms have been applied to optimize various parameters [51]. Nonetheless, metaheuristic algorithms are rarely used for Deep Learning (DL) parameter optimization problems [33]. The initial study to start this optimization utilizing metaheuristic algorithms proposed the merger of the Genetic Algorithm (GA) with DCNN [52]. In this method, the DCNN model is viewed as a chromosome in GA, and the DCNN process is chosen as cross-over and mutation on GA. In the cross-over phase, the DCNN model only modifies the biases and weights of the convolution layers. Rosa et al. suggested employing Harmony Search (HS) and some of its enhanced versions to fine-tune DCNN parameters in the field of handwriting and fingerprinting recognition [53].

Reference [54] described assigning pre-trained deep DCNNs using a Progressive Unsupervised Learning (PUL) technique. This technique is straightforward and serves as a valuable benchmark for feature selection. Due to the possibility of very noisy clustering results, this approach incorporates a selecting individual between the clustering and tuning processes.

An unsupervised DCNN topology selection method based on GA is suggested in reference [55] to optimize the parameters of image classification tasks. The main advantage of the presented methodology is its automatic method, which does not need any prior knowledge of the concept of DCNN. The main issue with this approach is that the GA’s chromosomes enlarge excessively in large DCNNs, which slows down the process. As a result, tweaking the DCNN hyperparameters is a difficult task when dealing with big DCNN-like image processing problems. Young [56] used GA to optimize a three-layer DCNN’s hyperparameter. Whenever the number of hidden layers is unknown, this optimizer is worthless. In order to find a good set of combinations, Reference [57] used a mutation-only optimization approach to develop the DL gradually. However, because the algorithm only considers mutations, this process is slow.

Due to the mentioned shortcomings, as well as the nature of image processing tasks, in which each dataset requires a particular structure with a different network depth [58], this paper introduces adaptive habitats for the BBO algorithm. This study derives its inspiration and basic technique from influential publications by Refs. [55,56]. However, the primary purpose of this study goes beyond the specific frameworks proposed in these earlier studies. The matter at hand pertains to a universal problem that is relevant to a common obstacle in the field of contemporary machine learning and artificial intelligence. This challenge involves the meticulous and proficient adjustment of intricate model parameters in order to improve the computational resilience and accuracy of performance.

BBO is a computational technique that draws inspiration from the notion of biogeography [59]. BBO emulates the migration patterns observed in species as a means to address optimization problems. Since its origin, multiple scholars have put forth several variations of BBO with the aim of improving its efficiency and flexibility. The objective of this analysis is to compile and examine these notable variations, emphasizing their distinct characteristics, practical uses, and enhancements in performance.

Enhanced Biogeography-Based Optimization (EBBO) [60] is an early adaption that incorporates an extra mutation mechanism into the conventional BBO algorithm. The mutation process is initiated subsequent to the migratory phase, with the primary objective of preserving diversity within the habitats. This process serves to impede early convergence and foster the development of global search skills. The usefulness of EBBO has been established in a range of benchmark optimization problems, exhibiting enhanced explorative capabilities compared to conventional BBO methods. Although EBBO supplied a supplementary mechanism for mutation, it had difficulties in parameter calibration, resulting in either excessive random searches or premature convergence. The mutation technique employed by the system was subject to criticism due to its lack of adaptability, which resulted in suboptimal performance when confronted with intricate or dynamic challenges.

The use of a Pareto optimality technique is a crucial feature of Multi-Objective Biogeography-Based Optimization (MOBBO) [61], which recognizes the need to address problems with multiple objectives. The system retains a collection of solutions, prioritizing the consideration of trade-offs among competing objectives. The critical advantage of MOBBO is its capacity to uncover a wide range of Pareto optimal solutions that are relevant in practical situations involving conflicting objectives, such as the distribution of resources and environmental planning. Although MOBBO has demonstrated efficacy in managing multiple objectives, it has encountered challenges in preserving a varied Pareto front, particularly in scenarios involving high-dimensional areas. Furthermore, there were instances where it exhibited inadequacies in achieving a satisfactory equilibrium between convergence and variety, resulting in less-than-ideal compromises in the proposed solutions.

The Discrete Binary Biogeography-Based Optimization (DBBBO) algorithm is a modified version of the traditional BBO algorithm [62]. Its purpose is to address binary optimization problems, which are particularly relevant in several domains, such as feature selection, scheduling, and network design. The expansion of the applicability of BBO to domains that involve discrete variable optimization is achieved by the adaptation of solution representation and migration operator to accommodate binary choice variables. The binary character of DBBBO imposed constraints that restricted its applicability solely to discrete situations. Additionally, the absence of a method to mitigate early convergence rendered it inadequate for tasks necessitating thorough exploration.

The incorporation of chaotic maps into the BBO algorithm, known as Chaotic Biogeography-Based Optimization (CBBO) [63], serves to augment the exploration capabilities, particularly in the early stages of the search process. The utilization of chaotic sequences in the context of CBBO is advantageous due to their sensitivity to beginning circumstances and ergodicity. These properties contribute to the promotion of diversity and the prevention of local optima traps. Consequently, CBBO is particularly well-suited for addressing problems that exhibit complex, multimodal landscapes. While the utilization of chaotic maps by CBBO has shown advancements in exploration, it does not consistently ensure convergence stability, hence resulting in outcomes that are difficult to anticipate. The technique also shows a deficiency in providing a well-defined criterion for selecting the most appropriate chaotic map in relation to a particular challenge, hence affecting its overall flexibility [64].

Hybrid Biogeography-Based Optimization (HBBO) is a novel approach that arises from the amalgamation of BBO with symbiotic organism search [65]. This integration is driven by the acknowledgment of the synergistic capabilities exhibited by these diverse algorithms. Hybrid models, which aim to achieve a balance between exploration and exploitation, have demonstrated efficacy across diverse domains, exhibiting accelerated convergence rates and enhanced resilience when confronted with intricate optimization problems. The HBBO models, albeit exhibiting resilience, frequently encountered heightened computational intricacy as a result of the incorporation of several algorithms. The researchers also encountered difficulties in effectively managing the advantages of the integrated methodologies, occasionally resulting in incongruity in outcomes across various problem scenarios.

The Adaptive Biogeography-Based Optimization (ABBO) algorithm [66] incorporates adaptive mechanisms that dynamically modify parameters, such as mutation rates or immigration rates, in response to the ongoing search progress. The algorithm's versatility enables it to adjust to the dynamics of the challenge efficiently, ensuring a balanced approach between exploration and exploitation during the search process. ABBO demonstrates a high level of efficacy in dynamic contexts characterized by non-stationarity, wherein the optimization parameters have the potential to vary over time. The dynamic adjustment techniques implemented by ABBO were characterized by innovation, yet, they tended to be overly responsive to slight variations within the search space. As a result, this sensitivity led to undesired fluctuations in parameters and occasional deviations from the optimal pathways.

Quantum Biogeography-Based Optimization (QBBO) [67] incorporates techniques from quantum computing to introduce quantum bits in solution representation, hence augmenting the exploration of the solution space. The utilization of the superposition of states in QBBO enables the concurrent exploration of several potential solutions, hence offering a notable advantage in addressing optimization problems that are high-dimensional and complex. This advantage is particularly relevant in the context of machine learning and data analysis. The QBBO algorithm, however innovative in its exploration of many solutions, exhibited a high level of complexity and lacked user-friendly accessibility for common optimization tasks. The performance of the system was also significantly dependent on the accurate initialization of quantum bits, and it necessitated considerable computer resources.

Giri et al. introduced the Adaptive Neighborhood for Locally and Globally Tuned Biogeography-based Optimization Algorithm (ANLGBBO) [68], which aims to improve upon the traditional BBO algorithm. The strength of the study resides in its adaptive neighborhood mechanism, which enhances both local and global search capabilities, resulting in a more nuanced equilibrium between exploration and exploitation. Nevertheless, one must acknowledge a constraint that arises in terms of the computational complexity of the algorithm and the absence of thorough compared analysis with other algorithms of the same era. These shortcomings give rise to inquiries over the accuracy and efficiency of its performance benchmarks.

In contrast, the Biogeography-Based Optimization Algorithm with Momentum Migration and Taxonomic Mutation (BBOMMTM) [69] incorporates innovative principles of momentum migration and taxonomic mutation with the objective of mitigating premature convergence and preserving genetic diversity within habitats. Although the new approach demonstrates enhanced proficiency in managing intricate multimodal functions, one drawback of the methodology is its propensity to add randomness and unpredictability to the solution, hence diminishing its reliability for specific applications.

The Improved Biogeography-Based Optimization Algorithm Based on Hybrid Migration and Dual-Mode Mutation Strategy (MDMSBBO) [70] offers a distinct approach by incorporating a dual-mode mutation technique alongside a hybrid migration strategy, hence augmenting its capacity for global optimization. The primary strength of this article is in its capacity to effectively address a diverse array of optimization problems, hence substantially mitigating the likelihood of premature convergence. Nevertheless, this approach results in an augmented parameter set, rendering the algorithm more vulnerable to the problem of parameter sensitivity. Consequently, its applicability across diverse situations or for users with differing degrees of proficiency may become more intricate.

### 1.1 Research gaps and motivation for the AHBBO

The restrictions mentioned above emphasize the ongoing requirement for a solution that is more equitable, economical in its use of resources, and adaptable to a variety of optimization scenarios. The AHBBO is motivated by several critical deficiencies in the existing literature, namely in relation to adaptability in dynamic contexts, consistent convergence, effective exploration-exploitation balance, and computing simplicity.

The AHBBO is proposed as a solution to overcome the limitations of conventional BBO approaches. This study suggests the implementation of flexible habitat sizes and controlled mutation processes as potential solutions to address the problems of early convergence and inadequate exploration, which were commonly observed in previous models. The incorporation of varied habitat sizes in AHBBO enables the accommodation of a broader spectrum of issue structures, hence fostering variation within the search process and supporting improved adaptability to intricate, multimodal optimization environments.

Moreover, the distinctive methodology employed by AHBBO in determining mutation and immigration rates, which are influenced by the quality of solutions, guarantees a search process that is more knowledgeable and sensitive to the surrounding context. The adaptability mentioned above not only serves to boost the speed of optimization but also guarantees a more efficient search that is essential for real-time applications and problems of significant scale.

In summary, the creation of AHBBO can be attributed to its predecessors' combined limitations and unaddressed areas of research. The mentioned development in BBO represents a significant advancement aimed at enhancing the dependability, adaptability, and efficiency of the optimization tool. This upgrade is intended to address the various complexities encountered in modern real-world applications effectively. Prior to examining the empirical components, it is essential to clarify the precise research questions that this study aims to answer and provide a thorough justification for choosing AHBBO for this complex undertaking.

### 1.2 Objectives and research questions

The main focus of this research is on three critical inquiries aimed at exploring the possible effectiveness of AHBBO in the domain of DCNNs. The main objects are:

1. **Effectiveness of AHBBO:** Does the AHBBO demonstrate proficiency in effectively navigating the complex search space inherent in DCNNs for the purpose of optimizing hyperparameters efficiently?
2. **Comparative Performance Analysis:** In comparison to traditional optimization algorithms, this inquiry seeks to evaluate the performance of AHBBO in terms of dependability, efficiency, and accuracy.
3. **Real-world Applicability:** What are the potential wider ramifications of implementing the AHBBO technique in practical situations involving DCNNs, such as complex image recognition tasks or sophisticated anomaly detection in large datasets?

This work seeks to address these inquiries in order to validate the theoretical effectiveness of AHBBO and compare its practical feasibility with conventional paradigms. As a result, it intends to bridge a significant gap in current literature.

### 1.3 Rationale for AHBBO's selection

The process of optimizing hyperparameters in DCNNs can be challenging due to the curse of dimensionality and the tendency to get trapped in local minima. The utilization of conventional methods, while prevalent, demonstrates significant inefficiencies when operating within these limitations, hence requiring a more resilient and adaptable strategy. The AHBBO system incorporates novel elements derived from biogeography-based optimization techniques. The algorithm demonstrates enhanced exploration and exploitation capabilities in search spaces with a high number of dimensions, thanks to its adaptive habitat selection and nuanced movement operator.

Several qualities make AHBBO a desirable candidate for this application:

• **Adaptability in Dynamic Landscapes:** The importance of adaptability in dynamic landscapes is exemplified by AHBBO, which possesses the ability to modify its methods dynamically. This characteristic is particularly crucial when confronted with intricate neural network designs that consist of multiple layers of hyperparameters that interact with each other.
• **Resource Efficiency:** Preliminary findings suggest that AHBBO can attain equivalent or higher outcomes while utilizing fewer computational resources compared to its counterparts. This characteristic is of utmost importance in the resource-intensive domain of deep learning.
• **Theoretical Soundness**: The theoretical foundations of AHBBO propose that it possesses a more remarkable ability to withstand premature convergence, a prevalent problem encountered by conventional optimizers when dealing with intricate, multimodal search environments that are inherent to DCNNs.

Compelling factors, including early results and theoretical rationale, support the selection of AHBBO for this study. This choice is based on the hypothesis that AHBBO has the potential to establish a novel paradigm in hyperparameter optimization for DCNNs.

Therefore, this concept is applied to various image classification problems with different dimensions. In the canonical BBO, the habitat’s length is first determined by the number of optimization parameters and remains constant until the end of the optimization process. This approach does not efficiently tackle the problems with large numbers of parameters in complex optimization problems such as digital image classification using DCNN. Therefore, we propose a substitute approach based on an adaptive length habitat strategy. The BBO initiates with a short habitat and then determines a habitat with the optimum habitant numbers. The AHBBO is first applied to 43 benchmark optimization problems, including 23 well-known standards, 20 CEC 2005 benchmark functions, and ten problems of the CEC06-2019 100-digits challenge to investigate the efficiency of AHBBO compared to eight benchmark optimization algorithms as well as canonical BBO. Then, we apply the AHBBO to tune the DCNN’s hyperparameters for the sake of digital image classification problems. In this regard, we utilize twenty well-known image classification datasets. The significant contribution of this paper is as follows:

• Proposing a unique approach for the optimization of DCNN hyperparameters using metaheuristic algorithms.
• Applying BBO for optimizing DCNN hyperparameters for the first time.
• Defining the adaptive variable length habitant BBO concept customized for DCNN with various depths (AHBBO).
• Incorporating immigration probability in AHBBO and practical model evaluation leads to an efficient discovery process.
• Evaluating AHBBO's efficiency with 53 test optimization strategies.
• Investigating the efficiency of DCNN_AHBBO using nine challenging image classification problems compared to 23 well-known image classifiers.

The paper is organized in the following manner. Section 2 provides a concise overview of the background information pertaining to BBO. The proposed methodology is presented in Section 3. The outcomes and evaluation of the experiment are reported in Section 4. Ultimately, the concluding section, Section 5, provides the final analysis and summary of the research findings.

## 2 Related terminology: Biogeography-Based Optimization Algorithm

Simon introduced the BBO optimizer, which takes into account the spatial and temporal distribution of biological entities [47]. This approach utilizes habitats as individuals to optimize a given problem, where each habitat consists of variables referred to as habitants. Each habitat's fitness value, the HSI, assesses whether it is suitable for a specific individual or species. BBO uses migration, mutation, and selection to improve each habitat's HSI. BBO initially creates a collection of habitats with *n* distinct residents in each habitat at randomness. The quantity n in a DCNN is the same as the amount of hyperparameters. It should be emphasized that the rates of emigration, migration, and mutations vary according to the surroundings. Two migration rates, the emigration rate $\left(\mu_{k}\right)$ and immigration rate $\left(\lambda_{k}\right)$, are used in this algorithm's implementation. The emigration rate $\left(\mu_{k}\right)$ is high for habitats with a high HSI score, while the immigration rate $\left(\lambda_{k}\right)$ is low for those with a low HSI score. Eqs. (1), (2) describe these two coefficients:

(1) $$\mu_{k}=E\times \left(\frac{k}{N}\right)$$

(2) $$\lambda_{k}=I\times \left(1-\frac{k}{N}\right)$$

The equations presented in this context involve several variables. Specifically, the variable "*k*" denotes the population size, "*I*" denotes the highest rate of immigration, "*E*" signifies the highest rate of emigration, and "*N*" denotes the maximal population capacity of the habitat. The mutation coefficient, as the third component of the BBO, serves to augment the system's capacity for exploration. This coefficient is defined by the following Eq. (3):

(3) $$m_{k}=m_{\max}\times \left(1-\frac{P_{k}}{P_{\max}}\right)$$

Where $m_{\max}$ indicates the mutation’s initial value. $P_{k}$ denotes the *k*th habitat mutation probability, and $P_{\max}$ can be formulated as Eq. (4).

(4) $$p_{\max}=\mathrm{argmax}\left(p_{k}\right),k=1,2,...,N$$

## 3 Proposed methodology

When using meta-heuristic methods to tune hyperparameters, such as those found in well-known benchmark references [56,57], two common shortcomings arise. Firstly, having a DCNN that is too small can result in underfitting. Secondly, having a DCNN that is too large can lead to overfitting.

These two issues can only be addressed by starting small and gradually increasing the size of the DCNN. As a result, we suggest an AHBBO for optimizing DCNN hyperparameters. It originates from a simple DCNN structure and subsequently grows on top of the simple structure. AHBBO leverages migration coefficients to accelerate the process while still preserving valuable resources (i.e., small DCNN).

### 3.1 Adaptive habitat BBO

**Fig. 1:**
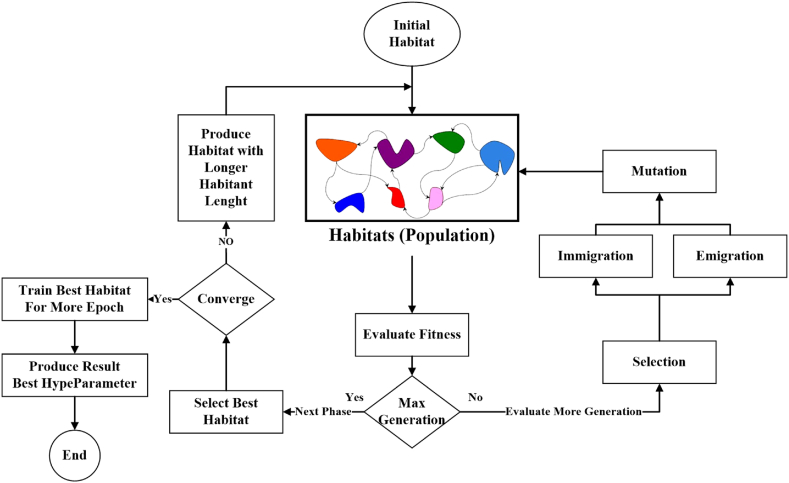
The general workflow for the AHBBO.

Canonical BBO requires a specific length of habitat. An AHBBO is more appropriate for our scenario because different DCNNs have different numbers of layers, and more hyperparameters become necessary as the DCNN gets deeper. In the AHBBO, habitat comprises solutions that specify a DCNN’s hyperparameter configuration. The upcoming section presents a technique for encoding hyperparameter settings within the habitat. Fig. 1 provides an overview of the complete AHBBO process. Also, the pseudocode of the proposed algorithm is shown in Algorithm 1. In order to create the initial population, two Convolutional Layers (CLs) are randomly chosen as hyperparameters (habitat). Then, according to their degree of appropriateness, the individuals present in the settings are evaluated and categorized. The fitness value is a term used to describe the effectiveness of the DCNN derived from the validation set.

Algorithm 1 Adaptive Habitat Biogeography-Based Optimization (AHBBO)

**Table:** 

**Input**: Initial parameters (population size, mutation rate, migration rate.), DCNN configuration details
**Output**: Optimized hyperparameters for the deep CNN
1: **procedure AHBBO**
2: Initialize the population with random habitats (each habitat represents a potential solution)
3: Determine the HSI (Habitat Suitability Index) for each habitat based on DCNN performance
4: **while** not reaching the stopping criteria, do
5: **for** each habitat in the population, do
6: Calculate the immigration and emigration rates based on HSI
7: **end for**
8: Perform migration operations using the calculated rates to update habitats
9: **for** each habitat in the population, do
10: Apply mutation based on the mutation rate
11: Update the HSI for the mutated habitat
12: **end for**
13**:** **Select the** habitats with the best HSI to form the new population for the next generation
14: **end while**
15: Adapt the habitat structure for a more complex DCNN (if the DCNN gets deeper)
16: **for** each new layer in the deeper DCNN, do
17: Adjust the habitat length (number of hyperparameters) based on the new layer
18: Repeat the optimization process for the new, more extended habitats
19: **end for**
20: Evaluate the final habitats based on the performance of the corresponding DCNNs
21: Select the habitat (set of hyperparameters) corresponding to the best-performing DCNN
22: **Return** The optimal set of hyperparameters
23: **end procedure**

The more suitable habitats are chosen and carried over to the next generation. Once the AHBBO algorithm has generated multiple generations of two-layer DCNNs, it enters the next phase, which enables the inclusion of additional hyperparameters and layers. During this phase, the habitat length is increased, allowing for more inhabitants to reside within each habitat, and more complex DCNNs with additional layers become desirable.

### 3.2 Encoding scheme

**Fig. 2:**
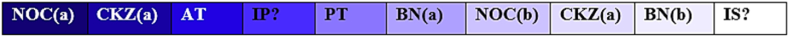
The first habitat.

**Fig. 3:**
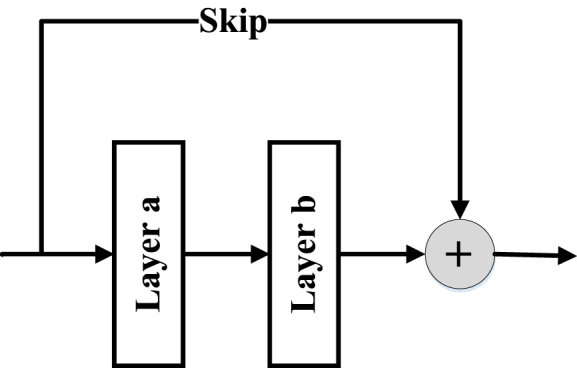
Skip connection (SC).

The AHBBO method encodes the hyperparameters of a DCNN model as habitats in BBO. In Phase 0, the habitats consist of two Convolution Layers (CLs) with associated hyperparameters, such as the size of convolutional windows, the number of output feature maps, and batch normalization. In addition, the habitats also encode the pooling type, Skip Connection (SC), and Pooling Layer (PL) inclusion in the block. The activation function type for the entire model is also specified in Phase 0. Fig. 2 depicts the coding pattern for a structure with two CLs. Also, Skip Connection (SC) is shown in Fig. 3. In subsequent phases, AHBBO adds two additional CLs to Phase 0 and allows for the flexibility of adding one or two CLs in each stage. In each succeeding phase, the habitats lengthen, and the DCNNs observed by AHBBO sink deeper. A subset of the hyperparameter variables for the larger DCNN constructions in later phases is provided by the optimum DCNN architecture from Phase 0. The habitats for the DCNN models discovered in each phase contain yellow fields that represent hyperparameters discovered in the current phase and grey fields that represent hyperparameters discovered in prior phases.

### 3.3 Fitness of habitats

**Fig. 4:**
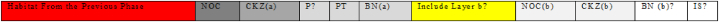
The encoding process for steps subsequent to Phase 0. The grey habitant shows the habitat from the previous phase. The current layer permits the addition of two CLs to the preceding layer. This encoding is achieved by adding an “IL b?” habitant with a yellow background color.

**Fig. 5:**
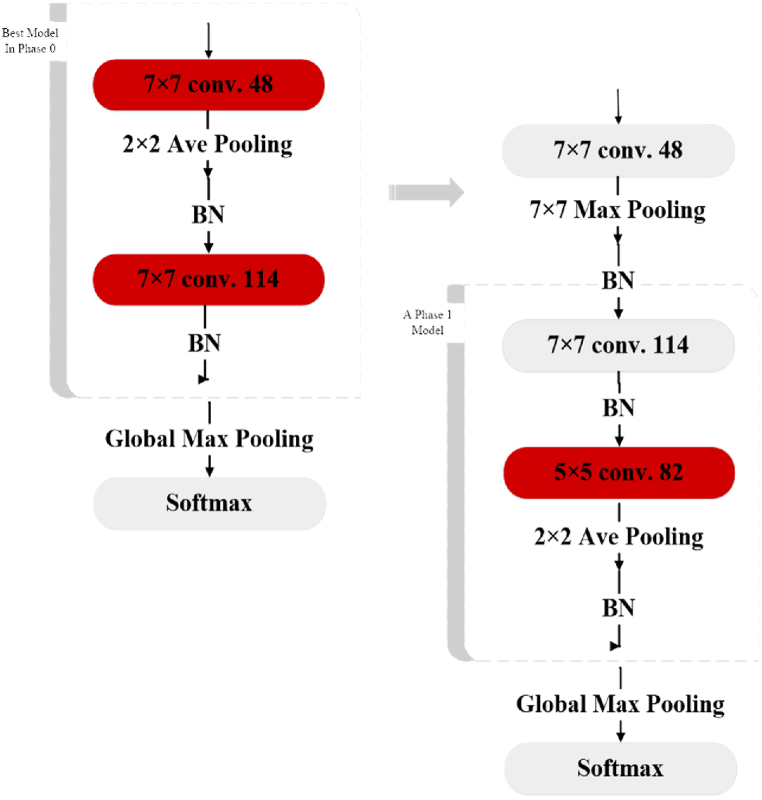
Every time a new phase starts, DCNN gets deeper.

**Fig. 6:**
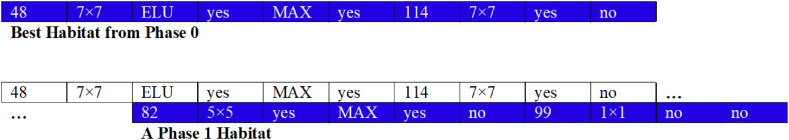
The habitats associated with DCNNs in Fig. 5 are related, and Phase 1 is an extension of the best habitat discovered in Phase 0.

**Fig. 7:**
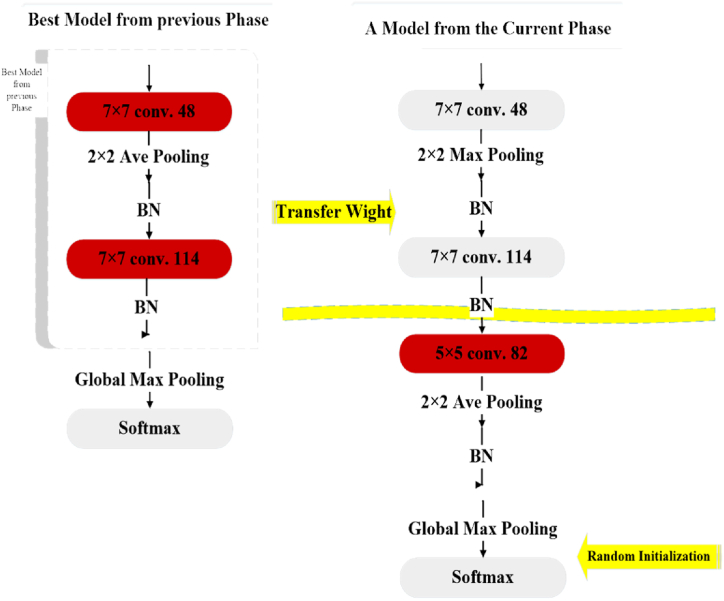
The map for weight initialization in deeper models.

In this method, habitat fitness is determined based on the accuracy of the validation data. The models can be trained until they converge. While evaluating models against a test set provides accurate results, it is a time- and energy-intensive process. In order to address this issue, DCNNs can be trained for a short period, typically a few epochs, and their average fitness can be estimated using the average fitness of other models. A DCNN that is fitter during the early training epochs performs better over all epochs. Five training epochs are the maximum number that can be experimentally employed to compare relative fitness. The encoding process for steps subsequent to Phase 0 is shown in Fig. 4, Fig. 5. Also, Fig. 6 shows the habitats associated with DCNNs in Fig. 5, in which Phase 1 is an extension of the best habitat discovered in Phase 0. Finally, the map for weight initialization in deeper models is shown in Fig. 7.

When there is a significant variation in the length of the DCNN structure, the evaluation based on this method can be biased. A deeper DCNN can exhibit analogs or perhaps have a worse accuracy rate than a shallower (e.g., 5) DCNN after only a few epochs of training. This problem is addressed by shifting weights from shallow DCNN to deeper DCNN. As the AHBBO project advances into its next stage, it delves into the study of more complex DCNNs, as depicted in the right portion of Fig. 7. Additionally, it enhances the performance of the most successful DCNN from the prior phase by incorporating additional layers, as illustrated in the left portion of Fig. 7. While some of the weights in the new DCNNs are mapped from the previous best DCNN, some are initialized stochastically, as opposed to all of them being randomly initialized. This approach makes use of already trained DCNNs, hence reducing training time. Since a portion of the deeper DCNN has already been trained, it is simple to train it for five epochs to evaluate its fitness.

## 4 Experimentation and discussion

In this part of the paper, we evaluate the suggested AHBBO and compare its results to those of established techniques. In this context, three different sets of test challenges are used:

1) Standard benchmark functions can be found in Ref. [71].
2) CEC2005 special session on real-parameter optimization [72], which are variants of the traditional benchmark problems that have been rotated, shifted, enlarged, and combined. These benchmark optimization problems are the most challenging of the traditional benchmark optimization issues.
3) The third group contains information on each of the ten test functions from the CEC06 2019 100-Digit challenge. The best value for all functions is 1.000000000 [73].

The outcomes of AHBBO are therefore contrasted with eight well-known optimization algorithms, namely the Ant Lion Optimizer (ALO) [74], Slime Mould Algorithm (SAM) [75], Levy’ Flight GWO (LGWO) [76], PSO [77], Krill Herd (KH) [78], WOA [79], HGSO [80] as well as original BBO. The parameter values of these methods are listed in Table 1. A Windows 10 PC with a 3.8 GHz Intel Core i7 processor, 32 GB of RAM, and Matlab R2021a was used for the research. The tables of results are produced after 30 iterations of the computations, and they include the average and standard deviation (AVE and STD). It is important to note that the most critical findings are bolded. According to Derrac et al. [81], nonparametric statistical assessment is essential in addition to statistical investigations to increase the effectiveness of meta-heuristic algorithm evaluation.

**Table 1:** Initial values and parameters for utilized algorithms.

Algorithm	Parameter	Value
BBO	Step size	1
	Immigration probability bounds	[0, 1]
	Habitat modification probability	1
	Maximum (*E*) and (*I*)	1
	The probability of mutation	0.005
SMA	$\vec{vc}$	[-1,1]
	*w*	[0.4, 0.9]
LGWO	*a*_0_	2
	*β*	U(0,1.5)
	*p*	U(0,2)
ALO	w	[2,6]
	*k*	500
PSO	c_1_	1.5
	c_2_	1.5
KH	V_f_	0.02
	Max(D)	0.005
	Max(N)	0.01
WOA	α	[1.5 0]
	β	0.95
HGSO	Cluster number	4
	M_1_	0.2
	M_2_	0.1
	α	1
	*β*	1
	K	1

**Fig. 8:**
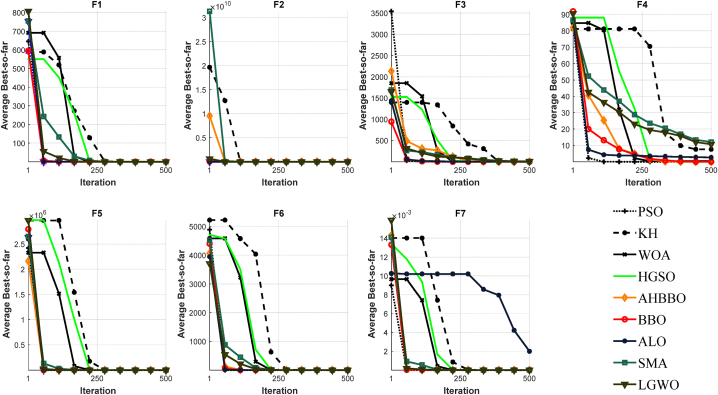
The plot of AHBBO and benchmarks on the unimodal sets.

**Fig. 9:**
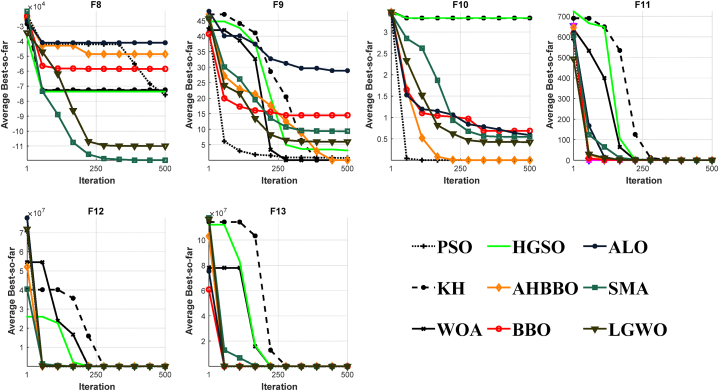
The plot of AHBBO and benchmarks on the multimodal sets.

**Fig. 10:**
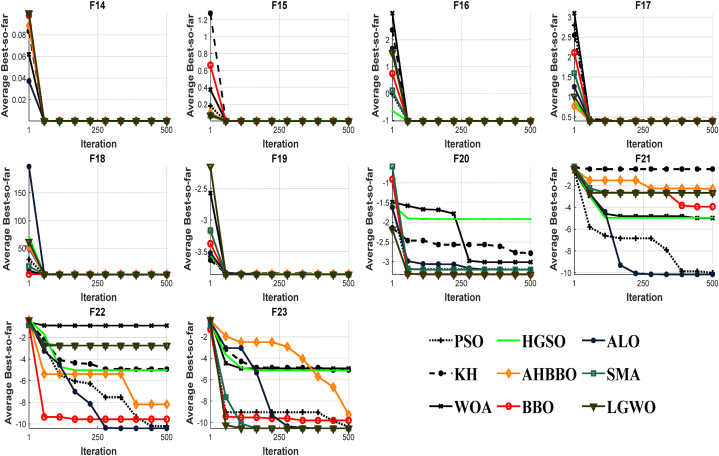
The plot of AHBBO and benchmarks on the fixed multimodal sets.

**Fig. 11:**
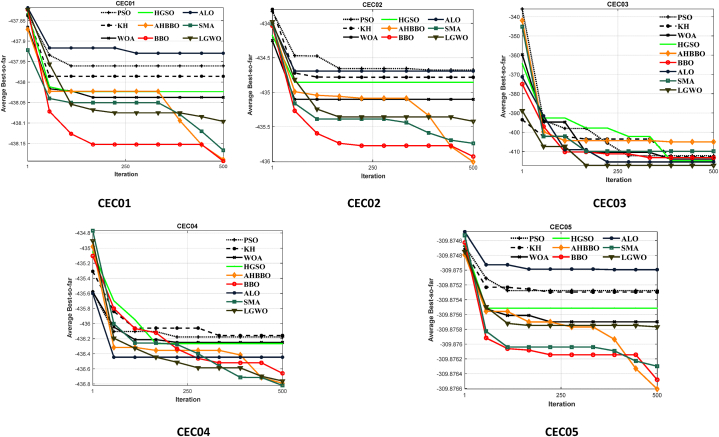
The plot of AHBBO and benchmarks on CEC2005 unimodal sets.

**Fig. 12:**
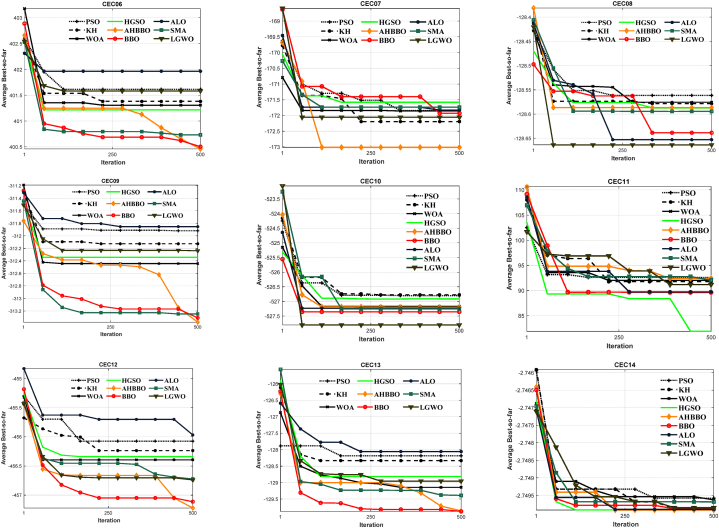
The plots of AHBBO and benchmarks on CEC2005 multimodal sets.

**Fig. 13:**
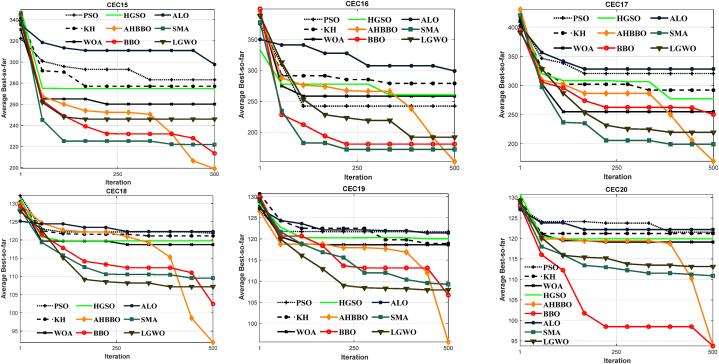
The plots of AHBBO and benchmarks on CEC2005 composite sets.

Fig. 8, Fig. 9, Fig. 10, Fig. 11, Fig. 12, Fig. 13 depict the results obtained from the experiments conducted. Table 2, Table 3, Table 4 provide the outcomes for the unimodal, multimodal, and fixed-dimensional testing functions, and Table 5, Table 6, Table 7 show the findings for the CEC2005 functions. The STD and AVE are used to assess the technique's robustness against local minimums. At a 5% level of significance, we utilized the nonparametric Wilcoxon's rank-sum test [81] to evaluate AHBBO's effectiveness to that of other benchmarking meta-heuristic techniques. The tables provide the AVE and STD values, as well as the p-values from the test. In cases where a method was compared against itself, the notation "Not Applicable" (N/A) was used [82].

**Table 2:** The findings of unimodal benchmarking sets.

Technique		*F*_*1*_	*F*_*2*_	*F*_*3*_	*F*_*4*_	*F*_*5*_	*F*_*6*_	*F*_*7*_
**ALO**	Ave	2.59E-10	1.84241E-06	6.06847E-10	1.36061E-08	**0.35118**	2.563E-10	0.004292492
	Std	1.72E-10	6.69E-07	6.42E-10	**1.81E-09**	**0.1100**	1.563E-02	0.005089
	p-value	0.0001	0.0001	0.0001	0.0001	**N/A**	0.0001	0.0001
**SMA**	Ave	1.08E-64	0.07292	0.4891	2.6088	25.0799	0.816579	0.0154
	Std	0.0000	0.0938	4.1144	0.8389	12.1226	0.000126	0.0012
	p-value	0.00797	0.00797	0.0047	0.0047	0.0001	0.0001	0.0047
**LGWO**	Ave	6.63E-27	7.25E-16	3.33E-05	5.61E-07	27.1812	4.59441	0.0014
	Std	6.34E-05	0.029014	79.14958	1.36884	10. 2754	1.12243	0.0092
	p-value	0.0001	0.033	0.024	0.0001	0.0001	0.0001	0.0001
**BBO**	Ave	6.87E-65	**2.19E-28**	1.401E-08	**1.4402E-12**	27.1546	0.2159	0.0011056
	Std	6.34E-12	0.00000035	0.0000221	0.00001991	0.0016991	0.0009889	0.541e-03
	p-value	0.00797	**N/A**	0.0047	**N/A**	0.0001	0.0001	0.0047
**PSO**	Ave	2.022E-12	6.8117E-12	3.8149E-05	1.4245E-02	27.1431	0.50114	0.00161
	Std	0.0011	0.0005	0.000171	0.0002914	0.00010121	0.0009804	0.001501
	p-value	0.0032	0.0032	0.0041	0.0046	0.0038	0.0021	0.0032
**KH**	Ave	6.1173E-48	0.0006	3.8819E-07	0.9313	58.5251	1.2803	3.3205
	Std	0.0000	0.00124	9.22e-03	0.000603	0.001109	0.001903	0.001102
	p-value	0.0032	0.0032	0.0036	0.00714	0.0045	0.0045	0.0078
**WOA**	Ave	2.8113E-51	0.0007	3.8819E-07	1.732E-04	20.8532	0.058577	0.10077
	Std	0.0000	8.4022e-05	0.0013834	0.00052112	0.00010311	0.00026976	7.668e-05
	p-value	**0.6584**	0.00797	0.0049	0.00797	0.0047	0.0057	0.00797
**HGSO**	Ave	9.2738E-25	0.05876	3.8819E-07	1.85E-07	39.7043	**1.0706E-13**	0.072983
	Std	0.1254	0.001456	0.00011335	0.0030542	0.00020054	**2.12E-09**	0.0004748
	p-value	0.00797	0.0057	0.0049	0.0049	0.0057	**N/A**	0.00797
**AHBBO**	Ave	**5.8443E-67**	6.8737E-17	**3.8819E-11**	1.4583E-06	27.1736	1.502E-10	**0.0011**
	Std	**0.0000**	**3.54E-07**	**234E-11**	0.00029769	0.00010131	2.12E-03	**0.0015441**
	p-value	**N/A**	**0.157**	**N/A**	0.0057	0.0049	0.0049	**N/A**

**Table 3:** Ave, Std, and p-values of multimodal sets.

Algorithm		F_8_	F_9_	F_10_	F_11_	F_12_	F_13_
**ALO**	Ave	−5610.1	80.88997	2.00733	0.0063	0.2708	0.3297
	Std	438.7	20.29005	0.77081	0.0039	0.3287	0.0736
	p-value	0.0001	0.0024	0.0157	0.0057	0.0049	0.0049
**SMA**	Ave	−12569.4	0.0001	8.882E-16	**0.0000**	0.0011	0.0004
	Std	**0.1**	**0.0000**	0.00000	**0.0000**	0.0014	0.0006
	p-value	**0.5214**	**0.5214**	0.0149	**0.0549**	0.0057	0.0001
**LGWO**	Ave	−6458.7	0.0984	0.0000	0.0006	0.0411	0.0169
	Std	859.4	0.0341	0.0000	0.0004	0.0047	0.0106
	p-value	0.0057	0.0057	0.0537	0.0049	0.0057	0.0045
**BBO**	Ave	−852.3897	1.1596	2.0019	0.0003	0.0270	0.0063
	Std	17.6746	0.3010	0.1745	0.0002	0.02196	0.0027
	p-value	0.0457	0.0057	0.0457	0.0057	0.0049	0.0049
**PSO**	Ave	−1055.9085	0.0417	0.8658	0.09521	0.0025	0.0001
	Std	11.5861	0.0104	0.0767	0.01389	0.0002	0.0001
	p-value	0.0014	0.0025	0.0249	**0.6123**	0.0471	**0.2140**
**KH**	Ave	−858.3064	1.5091	2.0066	0.0007	0.0087	0.0005
	Std	21.4912	0.8053	0.1336	0.0000	0.0036	0.0004
	p-value	0.0074	0.0354	0.0457	0.0049	0.0049	0.0001
**WOA**	Ave	−1045.6876	0.0007	0.9929	**0.0000**	0.0009	0.0008
	Std	17.2952	0.0003	0.1100	**0.0000**	0.0069	0.0054
	p-value	6.39e-05	**0.5214**	0.0457	**0.5274**	0.0425	0.0049
**HGSO**	Ave	−1045.6623	0.5007	0.9284	**0.0000**	0.0021	0.0051
	Std	15.5351	0.1731	0.1471	**0.0000**	0.0085	0.0004
	p-value	0.0074	6.39e-05	0.0457	**0.6123**	0.0424	0.0062
**AHBBO**	Ave	−1045.6623	**0.0000**	**1.92E-17**	**0.0000**	**0.0001**	**0.0000**
	Std	15.5351	**0.0000**	**1.01E-08**	**0.0000**	**0.0002**	**0.0000**
	p-value	0.0014	**N/A**	**N/A**	**N/A**	**N/A**	**N/A**

**Table 4:** Ave, Std, and p-values of fixed multimodal sets.

Algorithm		F_14_	F_15_	F_16_	F_17_	F_18_	F_19_	F_20_	F_21_	F_22_	F_23_
**ALO**	Ave	2.0411	0.00031	−1.03163	0.3999	3.000028	−3.86254	−3.18654	−10.1511	−10.4015	−10.5343
	Std	1.2007	0.00062	−1.03163	0.3997	3	−3.86211	−3.1156	−9.14077	−8.58441	−8.55887
	p-value	0.0057	0.0057	0.0047	0.0049	0.0057	0.0045	0.0047	0.0054	0.0047	0.0011
**SMA**	Ave	1.0412	0.000354	−1.03162	0.3989	3.000021	**−3.86112**	−3.29622	−10.1519	**−10.4025**	−10.5343
	Std	0.2527	0.000632	−1.03162	0.3979	3	**−3.86112**	−3.27011	−9.14045	**−8.58455**	−8.55893
	p-value	0.0057	0.0057	0.0047	0.0049	0.0057	**0.521**	0.0047	0.0054	**0.521**	**0.521**
**LGWO**	Ave	2.0421	0.000345	−1.03162	0.3998	3.000022	−3.86263	−3.28654	−4.1514	−5.4015	−10.5343
	Std	2.8541	0.000611	−1.03162	0.3978	3	−3.86278	−3.25056	−3.14015	−4.58441	−8.5545
	p-value	0.0057	0.0057	0.0047	0.0049	0.0057	0.0045	0.0047	0.0054	0.0047	0.0011
**ChOA**	Ave	1.0321	0.000302	**−1.03161**	0.3981	3.000028	−3.86244	−3.2899	−4.1524	−10.4015	−10.5223
	Std	1.0012	0.000605	**−1.03161**	0.3971	3	−3.86278	−3.2599	−3.14115	−8.58441	−8.55899
	p-value	0.0057	0.0057	**0.511**	0.0049	0.0057	0.0045	0.0047	0.0054	0.0047	0.0011
**PSO**	Ave	1.0021	0.000311	−1.03163	0.3985	3.000026	−3.86261	−3.29654	**−10.1529**	−10.4015	−10.5323
	Std	0.0799	0.000612	−1.03163	0.3985	3	−3.86278	−3.25111	**−9.14199**	−8.58441	−8.55899
	p-value	0.0057	0.0057	0.0537	0.0049	0.0057	0.0045	0.0047	**N/A**	0.0047	0.0011
**KH**	Ave	1.042493	0.000332	−1.03162	0.3981	3.000026	−2.86263	−3.28274	−2.1528	−6.4015	−7.5343
	Std	0.5217	0.000655	−1.03162	0.3981	3	−3.86278	−3.25001	−1.14019	−6.58441	−7.5522
	p-value	0.0057	0.0057	0.0537	0.0049	0.0057	0.0045	0.0047	0.0054	0.0047	0.0011
**WOA**	Ave	1.0124	0.000331	−1.03162	0.3981	3.000025	**−3.86112**	−3.28786	−10.1530	−1.4015	−10.5753
	Std	0.456	0.000605	−1.03161	0.3981	3	**−3.86112**	−3.25851	−9.14075	−1.58441	−8.55899
	p-value	0.0057	0.0057	0.0537	0.0049	0.0057	**N/A**	0.0047	0.0054	0.0047	0.0011
**HGSO**	Ave	1.0009	0.000344	−1.03162	**0.39801**	3.000025	−3.86258	−1.992	−5.1532	−6.4015	−6.5343
	Std	1.0021	0.000666	−1.03161	**0.39801**	3	−3.86278	−3.25147	−4.14096	−5.58441	−5.5511
	p-value	**0.1457**	0.0057	0.0537	**N/A**	0.0057	0.0045	0.0047	0.0014	0.0047	0.0011
**AHBBO**	Ave	**1.0008**	**0.000301**	**−1.03161**	**0.39801**	**3.000021**	**−3.8612**	−3.0004	−3.1532	−9.4025	−10.5343
	Std	**0.0012**	**0.000611**	**−1.03161**	**0.39801**	**3**	**−3.86112**	−3.0004	−2.14096	−8.58455	−8.55779
	p-value	**N/A**	**N/A**	**N/A**	**0.549**	**N/A**	**0.555**	0.0047	0.0056	0.0044	0.0011

**Table 5:** Ave, Std, p-values of CEC2005 unimodal sets.

Algorithm		CEC_01_	CEC_02_	CEC_03_	CEC_04_	CEC_05_
**ALO**	Ave	−432.3103	**−437.1952**	−412.1990	−432.2113	−297.2442
	Std	39.0010	**38.1702**	29.6999	25.0119	27.3331
	p-value	0.0452	**N/A**	0.0307	0.0023	0.0057
**SMA**	Ave	−437.355	−436.6711	**−439.3113**	−437.2155	−308.2301
	Std	33.2467	44.0971	**22.1962**	23.2911	23.0105
	p-value	0.0057	0.0057	**0.5432**	**0.574**	0.0041
**LGWO**	Ave	−433.0022	−432.9843	−415.3001	−435.8112	−307.6217
	Std	32.1211	42.3992	32.1996	24.0004	22.9852
	p-value	0.0057	0.0057	0.0037	0.0059	0.0045
**BBO**	Ave	−437.3007	−435.1116	**−439.1122**	−433.3113	−302.1144
	Std	37.6123	45.9821	**22.1110**	23.1992	25.8950
	p-value	0.0421	0.0074	**N/A**	0.0057	0.0049
**PSO**	Ave	−437.2601	−436.8417	−403.1108	−436.99828	−309.1005
	Std	33.5111	40.0194	36.0984	23.9987	20.3214
	p-value	0.5471	0.6541	0.0021	0.6123	0.6251
**KH**	Ave	−428.2964	−434.5111	−408.0901	−430.1107	−305.2217
	Std	41.4981	42.8993	32.1300	28.1981	21.2111
	p-value	0.00747	0.0354	0.0057	0.0049	0.0049
**WOA**	Ave	−437.6806	−436.4127	−407.1129	**−435.0094**	−308.1584
	Std	37.2112	40.1193	35.1952	**24.0998**	20.1069
	p-value	0.0021	0.0001	0.0057	**N/A**	0.0425
**HGSO**	Ave	−437.6003	−436.5007	−430.9112	−435.0111	−308.1521
	Std	35.5451	40.1731	29.1002	24.0128	20.1005
	p-value	0.0471	6.39e-05	0.0057	0.0423	**0.0524**
**AHBBO**	Ave	**−439.6003**	**−437.9997**	−430.9112	−435.412	**−309.9521**
	Std	**31.1151**	**38.1731**	29.1002	25.0128	**18.1005**
	p-value	**N/A**	**0.0548**	0.0057	0.0423	**N/A**

**Table 6:** Ave, Std, p-values of CEC2005 multimodal sets.

Algorithm		CEC_06_	CEC_07_	CEC_08_	CEC_09_	CEC_10_	CEC_11_	CEC_12_	CEC_13_	CEC_14_
**ALO**	Ave	410.320	−170.32	−113.11	−314.22	−302.63	98.52	−453.54	−128.99	−273.24
	Std	31.001	31.441	0.554	0.095	0.077	0.3085	0.3874	0.5218	24.541
	p-value	0.0052	0.0052	0.0087	0.0047	0.041	0.0047	0.0021	0.0047	0.0057
**SMA**	Ave	400.31	−172.97	**−132.90**	−309.55	**−311.31**	92.941	−456.31	−127.88	−271.44
	Std	30.531	33.081	**0.167**	0.0452	**0.011**	0.3097	0.5263	0.5102	23.220
	p-value	0.0057	0.0042	**N/A**	0.0045	**N/A**	0.0042	0.0049	0.0109	0.0057
**LGWO**	Ave	403.01	−172.22	−127.52	−312.11	−309.11	92.321	−457.25	−128.99	**−276.56**
	Std	32.121	30.085	0.321	0.0521	0.087	0.3992	0.5213	0.5013	**20.005**
	p-value	0.0057	0.0052	0.0011	0.0011	0.0057	0.0057	0.0037	0.0059	**N/A**
**BBO**	Ave	402.39	−173.30	−130.25	−309.88	−309.82	92.564	−452.21	−128.65	−271.01
	Std	32.611	35.692	0.223	0.0618	0.0619	0.8974	0.4123	0.5009	25.895
	p-value	0.0421	0.0047	0.0421	0.0054	0.0421	0.0074	0.0049	0.0057	0.0049
**PSO**	Ave	401.11	−170.14	−127.42	−313.91	−309.14	92.654	−458.32	−128.88	−273.65
	Std	38.541	31.619	0.111	0.0014	0.0121	0.2154	0.5001	0.4963	23.141
	p-value	0.0011	0.0053	0.0071	0.0063	0.0042	0.0068	0.5221	0.5523	**0.5124**
**KH**	Ave	401.17	−171.11	−128.52	−311.66	−309.87	92.541	−458.39	−128.65	−273.88
	Std	31.001	31.018	0.2314	0.0514	0.0851	0.546	0.5486	0.4596	25.212
	p-value	0.0071	0.0041	0.0074	0.0052	0.021	0.0354	0.0057	0.0049	0.0049
**WOA**	Ave	400.00	−170.66	−128.23	−313.07	−309.33	92.741	−457.99	−128.00	−272.02
	Std	30.211	30.559	0.5236	0.0436	0.0245	0.514	0.5888	0.6532	25.037
	p-value	0.0421	0.0057	0.0021	0.0047	0.0047	0.0048	0.0057	0.0020	0.0052
**HGSO**	Ave	401.51	−173.22	−129.25	−313.54	−30938	**92.654**	−458.81	−129.01	−275.91
	Std	30.505	30.104	0.2091	0.0451	0.0214	**0.4169**	0.5880	0.5231	21.185
	p-value	0.0021	0.0047	0.0017	0.0042	0.0071	**N/A**	0.5357	0.0023	**0.521**
**AHBBO**	Ave	**397.11**	**−174.22**	−129.25	**−316.54**	−310.38	92.654	**−459.91**	**−129.93**	−276.56
	Std	**20.545**	**28.114**	0.2091	**0.0051**	0.0214	0.4169	**0.121**	**0.1831**	20.185
	p-value	**N/A**	**N/A**	0.0042	**N/A**	**0.452**	0.0065	**N/A**	**N/A**	**0.521**

**Table 7:** Ave, Std, and p-values of CEC2005 composite sets.

Algorithm		CEC_15_	CEC_16_	CEC_17_	CEC_18_	CEC_19_	CEC_20_
**ALO**	Ave	238.12	180.2413	200.5463	108.3114	140.8521	140.2145
	Std	42.5330	55.1992	59.3256	35.2145	20.0119	44.3214
	p-value	0.0028	0.0059	0.0047	0.0047	0.0065	0.0052
**SMA**	Ave	268.124	175.325	**190.124**	95.853	135.329	131.811
	Std	50.1122	57.5213	**59.5544**	25.0896	20.7752	43.1236
	p-value	0.0075	0.021	**N/A**	0.0041	0.0053	0.0047
**LGWO**	Ave	300.2514	290.1152	310.6523	124.7012	128.0011	129.254
	Std	52.1255	56.321	58.6954	24.1286	19.0544	43.2569
	p-value	0.0047	0.0047	0.0037	0.0047	0.0047	0.0075
**BBO**	Ave	290.54	289.1111	310.1122	106.9534	140.1254	129.8521
	Std	47.8453	57.2315	54.2189	25.5148	20.1321	41.2569
	p-value	0.0421	0.0074	0.0047	0.0049	0.0047	0.0049
**PSO**	Ave	295.29	170.90	280.4569	95.5214	98.7012	96.3258
	Std	32.2531	51.0123	52.1286	22.2153	19.2546	**40.2156**
	p-value	0.0052	0.0047	0.0047	0.0036	0.0145	0.0013
**KH**	Ave	263.2964	190.5221	238.0974	126.9987	133.2154	111.2584
	Std	51.5581	53.8113	55.2589	24.1284	20.6548	41.2593
	p-value	0.0047	0.0045	0.0057	0.0075	0.0051	0.0047
**WOA**	Ave	214.1284	186.2489	220.1463	114.1584	110.2358	108.2186
	Std	52.9865	56.1147	57.2581	25.1243	20.3214	41.8965
	p-value	0.0045	0.0075	0.0047	0.0025	0.0028	0.0425
**HGSO**	Ave	**210.5463**	170.8521	220.9112	95.1999	102.5124	108.8525
	Std	**50.1154**	54.2365	57.8965	25.1247	28.3254	42.3004
	p-value	**N/A**	0.0056	0.0057	0.0046	0.0026	0.0049
**AHBBO**	Ave	211.53	**167.8521**	191.91	**75.1999**	**92.5124**	**92.8525**
	Std	52.1154	**44.2365**	61.8965	**10.1247**	**18.3254**	42.3214
	p-value	**0.541**	**N/A**	0.047	**N/A**	**N/A**	**N/A**

For the CEC2019 100-Digit Challenge, each algorithm was run 50 times on each benchmark problem. The function grades were divided by Nc/25 for the twenty-five executions with the lowest assessment times [83]. Each challenge has a maximum possible score of 10, and the maximum possible score for the task is 100 if the highest 25 among the 50 runs for each of the Ten testing challenges correspond to 10 decimal places. The minimum achievable objective value for all test issues is 1.000000000 with a precision of ten digits. This scoring means that a score of 3, 0, or 5 is awarded to an algorithm if it produces results with objective values that are 1, 0, or 1, respectively. The results of the CEC2019 100-Digit Challenge are shown in Table 8.

**Table 8:** CEC06-2019 challenge’s results.

Algorithm	ALO	SMA	LGWO	BBO	PSO	KH	WOA	HGSO	AHBBO
**1**	**10**	**10**	**10**	**10**	**10**	**10**	**10**	**10**	**10**
**2**	**10**	**10**	**10**	**10**	**10**	**10**	**10**	**10**	**10**
**3**	**10**	7.16	5.44	**10**	6.12	2.04	5.44	5.44	**10**
**4**	2.04	6.12	**4.96**	1.16	2.04	2.04	**4.96**	**4.96**	4.16
**5**	10	10	1.72	10	10	1.16	10	1.72	**10**
**6**	1	1.04	1	**4.96**	1	**4.96**	2.06	1	1.96
**7**	4	2.38	**10**	1.04	1	**10**	**10**	**10**	1.24
**8**	**10**	**10**	**10**	**10**	**10**	7.16	**10**	**10**	**10**
**9**	**10**	7.16	**10**	**10**	**10**	10	**10**	**10**	**10**
**10**	7.16	1	1	10	1.16	1	4.16	2.06	**10**
**grade**	74.20	64.86	64.12	77.16	61.32	58.36	76.62	65.18	**77.36**

### 4.1 Unimodal optimization benchmarks

Based on the obtained results, it appears that the AHBBO optimization method has performed well in the evaluation of unimodal test functions. According to the findings in Table 2, AHBBO performed the best on average for the F1 to F3 and F7 functions while also tying for first place in other functions, as shown in Table 5 for CEC01 and CEC05. This result indicates that AHBBO is effective at exploiting the single optimum in unimodal functions and is competitive with other optimization methods in other types of functions.

Furthermore, the convergence curves shown in Fig. 8, Fig. 11 suggest that AHBBO has the fastest convergence rate among all benchmark functions. This trend means that AHBBO can quickly converge to the optimal solution compared to other optimization methods, which is a desirable trait in optimization algorithms.

The results in Table 2, Table 5 also indicate that AHBBO achieved substantial results compared to other baseline optimization methods. The use of p-values suggests that the differences between AHBBO and other optimization methods are statistically significant and not simply due to chance. This metric provides additional evidence of the effectiveness of AHBBO as an optimization method.

Overall, the results suggest that AHBBO is a promising optimization method that performs well in a variety of test functions. Further analysis could be conducted to examine the strengths and weaknesses of AHBBO compared to other optimization methods in more detail, as well as to investigate its performance on more complex and challenging optimization problems.

### 4.2 Multimodal optimization benchmarks

Table 3, Table 6 display the outcomes for multimodal functions, underscoring the notable rise in the occurrence of local optima, which intensifies with an increase in the number of dimensions. The outcomes in Table 3, Table 6 demonstrate that AHBBO performed well on average for most functions, except for a few, such as F8, CEC08, CEC10, CEC11, and CEC14. Nevertheless, AHBBO's results are comparable to other baseline algorithms, and the differences are statistically significant based on p-values.

In terms of convergence, Fig. 9, Fig. 12 reveal that AHBBO, WOA, and LGWO exhibit quick convergence, while other algorithms, such as ALO, PSO, and KH, show steady-state convergence, which may cause them to get stuck in local minima. Therefore, the optimal convergence behavior, characterized by an accelerated form, is associated with AHBBO, BBO, and SMA in that order, as shown in the figures.

Overall, the results suggest that AHBBO is a competitive optimization method for multimodal functions, with comparable performance to other baseline algorithms. However, further investigation could be conducted to explore the reasons for its superior convergence behavior and the limitations of AHBBO in solving certain functions. Additionally, more research could be conducted to test AHBBO on more extensive and more complex optimization problems.

### 4.3 The fixed-dimension benchmark functions’ result

Based on the given information, fixed-dimension test functions have fewer local optima compared to multimodal test functions. However, they cover a more extensive search space than conventional test functions. Table 4 indicates that the outcomes of all algorithms are similar for five test functions, except for AHBBO, which performs competitively across all functions and outperforms the competition in five of these functions. This improvement suggests that AHBBO is a promising optimization method for fixed-dimension test functions.

The convergence curves for the optimization algorithms on fixed-dimension functions are presented in Fig. 10, and all the algorithms show a near convergence rate. Nonetheless, AHBBO outperforms the other algorithms by a small margin. The observed commonalities in the acquired findings and converging tendency could potentially be attributed to the low-dimensional architecture of the test mentioned above functions.

Overall, the results suggest that AHBBO is a competitive optimization method for fixed-dimension test functions, and it outperforms the competition in several cases. However, further investigation could be conducted to explore the reasons for its superior performance and limitations in solving more complex optimization problems.

### 4.4 Composite optimization functions’ outcomes

The findings from the composite functions analysis indicate that AHBBO has remarkable performance, outperforming all composite functions except for CEC15 and CEC17. Composite functions are known to be highly challenging benchmarks for optimization algorithms as they require a balance of exploration and exploitation phases. They also have many local optimum solutions, which can be used to evaluate a benchmark algorithm's ability to avoid local optima.

As seen in Table 7, AHBBO exhibits an optimal balance of exploitation and exploration phases, resulting in a high rate of local optima prevention. This balancing behavior is due to the skip connection's unique convergence characteristic, which enables AHBBO to simultaneously reflect reasonable utilization, investigation, and local optimality avoidance capability.

Based on the findings presented in Fig. 13, it can be observed that the convergence rates of AHBBO on composite functions demonstrate a consistent intensification factor across all iterations. This accelerating property enables AHBBO to surpass other test algorithms after 250 iterations, showcasing its remarkable optimization capabilities. Overall, the results suggest that AHBBO is a highly effective optimization method for composite functions and demonstrates a remarkable balance of exploration and exploitation.

### 4.5 CEC06-2019 benchmark function results

Table 8 provides a summary of the scores assigned to each of the nine benchmark methods for each of the ten challenges. The comparison algorithms include ABO, ALO, BBO, DE, GWO, KH, PSO, WOA, and AHBBO. Among these algorithms, AHBBO, BBO, and WOA have the highest scores for nine functions. However, the experiments conducted suggest that BBO-based algorithms have difficulty addressing F4 and F7, possibly due to the high dimension of the problems, which increases the length of the chromosome.

AHBBO addresses the issue with F4 by adjusting the chromosome's length, and as a result, it achieves the best performance statistically for 30 of 53 test functions. Additionally, AHBBO produces highly competitive values for the other composite functions, making it a promising algorithm for solving optimization problems. These results suggest that AHBBO is a highly effective optimization method for various types of optimization problems, including both unimodal and multimodal functions, fixed-dimension functions, and composite functions.

### 4.6 Image classification experiments

Several experiments are conducted on well-known image classification problems to assess the effectiveness of the developed DCNN-AHBBO further. The results are compared to a few chosen most recent benchmark methods. In the following, these chosen datasets, peer competitors, and parameter settings of the DCNN-AHBBO are explained.

#### 4.6.1 Benchmark datasets

The effectiveness of the proposed DCNN-AHBBO model is assessed using nine widely used image classification datasets, which are tabulated in Table 9. Note that the size of images in these benchmarks is 28 × 28.

**Table 9:** The image datasets for classification problems.

Row	Dataset	# Training sample	# Test sample
1	Fashion [84]	50000	10000
2	Convex Sets (CS) [85]	8000	50000
3	Rectangle Images (RI) [85]	12000	50000
4	MNIST [86]	12000	50000
5	MNIST Basic (MB) [85]		
6	MRB [85]		
7	MBI [85]		
8	MRDBI [85]		
9	MRD [85].		

#### 4.6.2 Benchmark classifiers

For the studies, a selection of 23 widely recognized algorithms that demonstrate satisfactory classification errors were selected as baseline classifiers, which are listed in Table 10.

**Table 10:** Lists twenty-three widely recognized algorithms.

Datasets	Number	Name
Fashion Dataset	1	2C1P [87]
	2	GRU + SVM + Dropout [87]
	3	3C2F [87]
	4	2C1P2F + Dropout [87]
	5	3C1P2F + Dropout [87]
	6	MLP 256-128-64 [88]
	7	AlexNet [89]
	8	SqueezeNet-200 [90]
	9	VGG16 [88]
	10	GoogleNet [87]
Other Datasets	1	CAE-2 [91]
	2	TIRBM [92]
	3	PGBM + DN-1 [93]
	4	ScatNet-2 [94]
	5	PCANet-2 (softmax) [95]
	6	RandNet-2 [95]
	7	EvoCNN [96]
	8	LDANet-2 [95]
	9	DBN-3 [85]
	10	SVM + Poly [85]
	11	SVM + RBF [85]
	12	SAA-3, NNet [85]
	13	PCANet-2 [95]

The selection of parameters to employ is often determined based on established practices within the deep learning and evolutionary algorithms communities, together with consideration of the optimum dimension of one of the primary layers. The validation dataset is also created by randomly selecting 20% of the photos from the training dataset. Thirty separate runs are performed on the used datasets due to the stochastic nature of the intended DCNN-AHBBO model, and the average scores of the results are taken into consideration for comparisons.

#### 4.6.3 The analysis of results

**Fig. 14:**
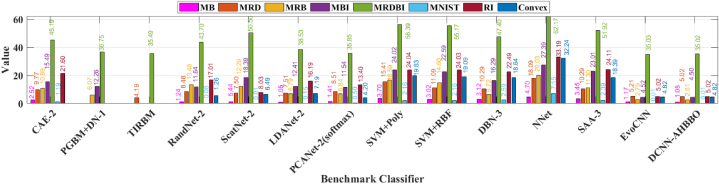
The classification errors for other datasets.

The findings from the experiments conducted on the Fashion dataset are outlined in Table 11. Additionally, Fig. 14 displays the results obtained from the MNIST-based, Rectangle, and Convex datasets. The final two rows of every table provide the average and best classification errors achieved by the DCNN-AHBBO method, while the remaining rows indicate the best classification errors attained by benchmark classifiers. It is essential to mention that the abbreviation "N/A" signifies that the provider did not provide the reported results.

**Table 11:** The classification errors for the Fashion dataset.

Models	Number of parameters	Error percentage	Number of epochs	P-value
**2C1P2F + Drouout** [16]	3.26 Million	8.39	300	0.001
**3C2F** [16]	N/A	9.29	N/A	0.002
**2C1P** [16]	100K	7.49	30	0.012
**GRU + SVM + Dropout** [16]	N/A	10.29	100	0.047
**3C1P2F + Dropout** [16]	7.15 Million	7.39	150	0.001
**AlexNet** [89]	59 Million	10.09	N/A	0.001
**GoogleNet** [97]	100 Million	6.29	N/A	0.024
**MLP 256**–**128**–**64** [88]	**40K**	10.01	**25**	0.001
**SqueezeNet-200** [98]	500K	10.01	200	0.012
**EvoCNN** [96]	6.69 Million	5.48	100	0.013
**VGG16** [88]	27 Million	6.49	200	0.011
**DCNN-Grid-Search** [99]	45K	8.44	30	0.001
**ANLGBBO** [68]	6.31 Million	6.11	100	0.002
**BBOMMTM** [69]	5.26 Million	5.69	100	**0.231**
**MDMSBBO** [70]	4.22 Million	6.02	100	**0.122**
**OPOA-ResNet-50** [100]	23.4 Million	7.04	100	0.012
**DCNN-AHBBO**	3.2 Million	**5.14**	100	N/A

Table 11 provides detailed information on the number of training epochs and parameters utilized in the Fashion dataset. At the same time, such information is not available for the benchmark datasets in Fig. 14. It is also stated that no data preprocessing was performed on the benchmark datasets or classifiers to ensure fair comparisons.

Overall, the results show that DCNN-AHBBO achieved competitive performance on all datasets, with the best classification error rates reported in several cases. For example, on the Fashion dataset, DCNN-AHBBO achieved the best classification error rate of 5.14%, while the best error rate reported by benchmark classifiers was 5.48%. Similarly, on the MNIST-based dataset, DCNN-AHBBO achieved the best classification error rate of 0.21%, while the best error rate reported by benchmark classifiers was 0.24%. These results suggest that DCNN-AHBBO has the potential to be a practical algorithm for solving image classification problems.

According to Table 11, the custom DCNN-AHBBO classifier is far superior to its eleven rivals. When contrasted to EvoCNN, the next-best classifier, the error rate is reduced by the suggested DCNN-AHBBO model to 5.14%. Additionally, compared to EvoCNN (6.69 M), VGG (27 M), and GoogleNet (100 M), DCNN-AHBBO's 3.2 M connection weights are significantly lower. It must be noted that these results are obtained with a much smaller number of epochs than other classifiers. The findings demonstrate that the developed DCNN-AHBBO model significantly improves DCNN accuracy on the Fashion dataset as well as the efficiency of architectural design.

Statistically, Fig. 14 shows that LDANet-2 achieve the highest results on the MB dataset, while DCNN-AHBBO is the best classifier across all 14 algorithms on the remainder of the datasets.

### 4.7 Search space

**Fig. 15:**
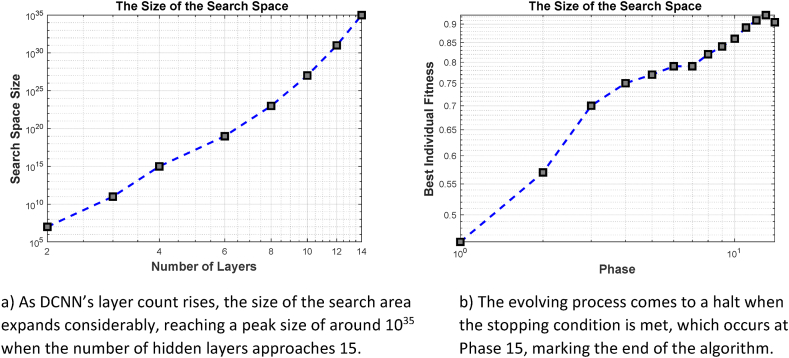
Stopping condition.

The search space for this experiment encompasses all possible individuals, including the Number of Output Feature Maps (NOFM) in each CL, whether to include an SC, the size of the Convolutional Filter (CF), the Pooling Type (PT), the including BN in a layer (NL) and Activation Function type (AF). It is imperative to include a significant amount of hyperparameters, including the size of the number of feature maps (NOFMs) and the size of the convolutional filters (CF), in every convolutional layer (CL) report, resulting in an increasingly prominent search space as the model becomes more intricate [101]. Consequently, the search process becomes more complex. Previous research has commonly neglected the inclusion of PT in their search domain. However, this study introduces the concept of several PLs coexisting within a single DCNN. Table 12 provides a comprehensive depiction of the search space pertaining to the conducted experiments. Additionally, Fig. 15a visually represents the exponential expansion of the search space in correlation with the escalation in computational complexity.

**Table 12:** Prefixed searching space.

**Abbreviation**	**Hyperparameter**	**Hyperparameter Choices**
**NOC**	Number of Output Channels	8, 16, 32, 64, 128, 256, 512
**CFZ**	Conv. Filter Size	1x1, 3x3, 5x5, 7x7, 9x9
**AFT**	Activation Function Type	ReLU, ELU, Tanh, SELU
**PT**	Type of Pooling Layer	Max, Average
**SC**	Skip Connection	Yes, No
**NL**	Number of Layers	≥2
**BN**	Batch Normalization	Yes, No

### 4.8 Stopping Condition

The progressive procedure is finished when the validation accuracy cannot be improved even after the addition of new layers. As soon as the best habitat’s fitness value in the next phase is less than a threshold value (e.g., 0.01 less than in the prior phases), the evaluation process is terminated, and we are restored to our original starting point. The process in Fig. 15b comes to a halt at the 15th phase. In Phase 15, the best habitat has a fitness of 0.9370, while in Phase 14, the best habitat has a fitness of 0.9233.

**Fig. 16:**
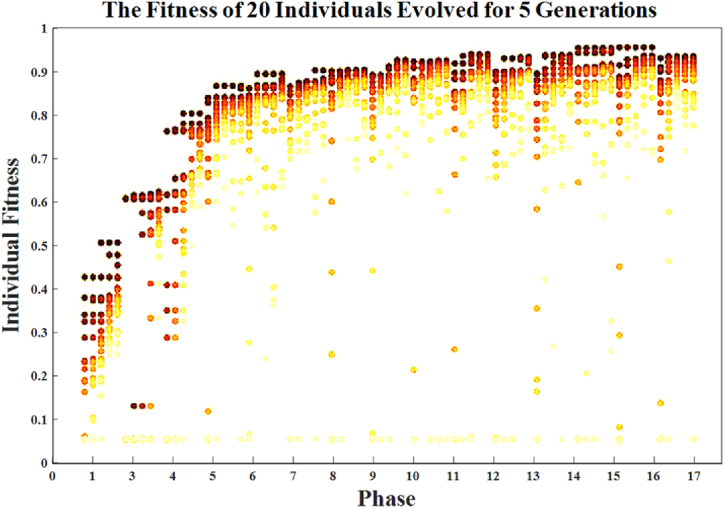
Twenty habitats produce an outcome that gradually changes for five generations during each phase.

The outcomes of gradually constructing 20 habitats over multiple generations for each phase are shown in Fig. 16. Each habitat goes through five epochs of training to estimate fitness. The evolution comes to an end in phase fifteen whenever the ending criteria are satisfied. A growing percentage of CLs are added with each phase. Individual accuracy initially rises rapidly with each algorithm improvement. The algorithm gets better initially, then gets worse. Our results suggest that increasing the number of convolutional layers can have a significant adverse effect on model performance, especially in cases when the structure is shallow. A small network might not be able to fix the issue. As both the layer count and network structure rise, the network's capacity increases.

Fig. 16 shows the outcome of twenty habitats that underwent progressive evolution over five generations for each phase; five epochs are used to train each habitat to approach the fitness function. Phase fifteen of the evolution occurs as soon as the termination criteria are satisfied. The layer count gradually grows with each succeeding phase. The precision of the habitats dramatically increases at the beginning of each fresh AHBBO phase. The final stage is when performance gradually deteriorates. Therefore, adding more layers can significantly enhance the DCNN’s performance, especially when the DCNN is somewhat shallow.

### 4.9 Discussion

The robustness and versatility of AHBBO are highlighted by the empirical evidence obtained through comprehensive testing on various benchmark functions. Nevertheless, it is imperative to analyze these data in order to comprehend the intricacies of the algorithm, identify any limitations in its potential applications, and identify potential areas for further investigation.

#### 4.9.1 The performance of AHBBO and its implications

The AHBBO algorithm exhibited remarkable competence, notably in the optimization of unimodal functions, a field that is frequently difficult for traditional algorithms. The novel exploitation strategy of the algorithm can be credited for its effective navigation of the search space and its capacity to converge towards the global optimum. This improvement is evident from its higher performance on functions F1 to F3 and F7. The architecture of AHBBO inherently promotes an extensive exploration within small neighborhoods, facilitating the ability to make exact adjustments, which is of utmost importance in landscapes characterized by a single dominant peak.

On the other hand, it is worth reflecting on the algorithm's decrease in performance observed in specific multimodal functions, particularly in F8 and CEC series tests such as CEC08 and CEC14. These functions, which exhibit multiple local optima, require a balance between exploration and exploitation. The inclination of AHBBO to prioritize extensive local searches may hinder its ability to explore globally, resulting in early convergence in such environments. This observation does not serve as a drawback but rather as a valuable understanding that influences the algorithm's scope of use.

To sum up, DCNN-AHBBO has demonstrated several significant strengths that set it apart in the landscape of hyperparameter optimization methods.

1. **Enhanced Performance:** DCNN-AHBBO has been shown to achieve superior classification accuracy on several benchmark datasets compared to traditional deep learning models. By efficiently navigating the hyperparameter search space, it identifies more refined and practical configurations that contribute to an observable increase in model performance.
2. **Efficiency in Optimization:** Unlike conventional grid and random search methods, DCNN-AHBBO significantly reduces the time required for hyperparameter tuning. By adaptively adjusting parameters during the optimization process, it converges to optimal solutions faster, thereby saving computational resources and time.
3. **Adaptability and Robustness:** The AHBBO component introduces a level of adaptability unseen in many standard optimizers. By adjusting its strategy based on the performance landscape, our approach maintains a balance between the exploration of new potential solutions and the exploitation of known practical configurations. This balance ensures a robust search process that is less susceptible to local minima and overfitting.
4. **Scalability:** Our approach scales well with the complexity and size of datasets. The adaptative mechanisms within AHBBO allow for efficient searching strategies regardless of the search space's dimensionality, making our method particularly suitable for complex real-world applications with extensive data.

#### 4.9.2 Study limitations and interpretational caution

Although our findings show promise, it is essential to note that they are derived from a controlled experimental setting. While this environment is diverse, it may not encompass all aspects of real-world optimization. The benchmark functions employed in this study, while methodologically robust, are conceptual constructions. The capacity of these models to accurately capture the complexity and unpredictability inherent in real-world situations, such as real-time data analytics, engineering design, or financial modeling, may be limited.

In addition, the comprehensive comparison study was, at times, limited by the inherent biases of some benchmark functions towards specific algorithmic tendencies. For instance, while AHBBO demonstrated a rapid convergence rate that provided it with a competitive advantage in unimodal testing, it is not always indicative of its dominance in complex, real-world issues that demand continuous exploration.

It is crucial to acknowledge that although statistical significance has been established based on p-values, this does not necessarily imply practical importance. Specific performance differences, while statistically significant, were minimal and should not be exaggerated.

To sum up, despite its notable advantages, it is imperative to acknowledge the constraints and potential drawbacks associated with the DCNN-AHBBO method:

1. **Parameter Sensitivity:** While AHBBO is adept at adapting its parameters, the initial setting of these parameters can significantly influence the efficiency and outcome of the optimization process. Finding the right balance requires a nuanced understanding of the problem space and may involve some trial and error.
2. **Computational Complexity:** Although more efficient than several traditional methods, DCNN-AHBBO involves complex computations, especially in the adaptive phase. This complexity can translate to higher computational costs, particularly for very large-scale problems or when operating with limited computational resources.
3. **Generalizability:** While our method has shown robust performance across multiple datasets, its effectiveness in other domains or radically different tasks remains an area for further exploration. The unique characteristics of different problems may require further tweaking and adaptation of the AHBBO mechanism.
4. **Over-Optimization Risk:** There is a potential risk of the model becoming overly tailored to the training data, especially with an extensive search for the 'perfect' hyperparameters. This over-optimization could potentially harm the model's ability to generalize to unseen data, thereby affecting its practical applicability.

#### 4.9.3 Comparative perspectives and prospects for the future

The analysis conducted indicates that the performance of AHBBO, while impressive, did not exhibit universal dominance. The efficacy of traditional approaches and other variants of the BBO algorithm has been demonstrated at various points, emphasizing that no single algorithm can be considered a universal solution. The presence of diverse performance outcomes underscores the need to utilize hybrid models or ensemble approaches that incorporate the capabilities of different algorithms and can adapt to the dynamic nature of a given challenge.

In anticipation of future developments, the incorporation of domain-specific knowledge into AHBBO has the potential to unleash novel opportunities. In the field of machine learning, the incorporation of embedding feature selection insights or model-specific heuristics has the potential to enhance the performance of AHBBO by making it more sensitive to the nuances present in the dataset. Furthermore, the adaptation of AHBBO for distributed computing settings has the potential to reduce calculation times significantly, which is a crucial improvement for real-time applications.

In summary, the AHBBO system represents a notable progression in the field of optimization, showcasing outstanding efficacy and adaptability. Nevertheless, the expedition does not conclude at this point. Acknowledging the constraints inherent in computational intelligence and deriving insights from them facilitates the emergence of novel advancements that have the potential to expand the frontiers of possibility.

## 5 Conclusion

In this paper, a new optimization algorithm called AHBBO is presented for fine-tuning the hyperparameters of DCNNs intended for image classification tasks. The suggested model was initially assessed using 23 standard functions, 20 CEC05 functions, and ten CEC06-2019 functions in terms of convergence rate, ability to avoid local minima, and the average of MSE. The investigations mentioned above confirm that applying the Adaptive habitat concept improves the general performance of conventional BBO. After approving the performance of AHBBO in numerical optimization functions, it was used to optimize the DCNN's default hyperparameters; the DCNN-AHBBO classifier was then evaluated against 23 well-known classification models on nine well-known image classification problems, including Fashion, CS, RI, MNIST, MB, MRB, MBI, MRD, and MRDBI. The results showed that the developed variable-length model could produce results that are quite comparable to those of these benchmark models.

Although AHBBO has several merits, the initial layer number and the number of searching agents (habitant for BBO) are challenging; therefore, several study directions for future work with the DCNN-AHBBO might be presented, including determining the number of initial layers, the number of searching agents, and other hyperparameters, which have not been considered. Future research directions can also consider underwater sonar target detection and categorization using the proposed model. Moreover, a potential contribution is the extension of AHBBO to handle multi-objective optimization problems. Future research directions may also explore the efficacy of utilizing chaotic maps, Levy flight, opposition-based updating, and dynamic habitat length in enhancing the performance of DCNN-AHBBO.

## Declaration

### Conflict of interest statement

The authors of the manuscript declare that they have NO affiliations with or involvement in any organization or entity with any financial interest.

## Data availability statement

The source code of AHBBO is available via https://uk.mathworks.com/matlabcentral/fileexchange/140871-adaptive-habitat-biogeography-based-optimizer.

## Funding statement

This work was supported by the Shandong Student Research Training Program (NO. D23024).

## CRediT authorship contribution statement

**Jiayun Xin:** Writing – review & editing, Visualization, Validation, Software, Project administration, Formal analysis. **Mohammad Khishe:** Writing – original draft, Supervision, Project administration, Conceptualization. **Diyar Qader Zeebaree:** Software, Investigation, Formal analysis, Data curation. **Laith Abualigah:** Writing – review & editing. **Taher M. Ghazal:** Writing – review & editing.

## Declaration of competing interest

The authors declare that they have no known competing financial interests or personal relationships that could have appeared to influence the work reported in this paper.
